# Prognosis of Penile Squamous Cell Carcinoma and Extramammary Paget Disease in Japan: An Analysis of Nationwide Hospital-Based Cancer Registry Data

**DOI:** 10.3390/cancers18142242

**Published:** 2026-07-13

**Authors:** Keisuke Sano, Shuhei Suzuki, Shuya Kandori, Reo Takahashi, Bunpei Isoda, Ryota Yanagihashi, Kazuki Hamada, Kozaburo Tanuma, Satoshi Nitta, Kosuke Kojo, Masanobu Shiga, Yoshiyuki Nagumo, Atsushi Ikeda, Takashi Kawahara, Akio Hoshi, Bryan J. Mathis, Ayako Okuyama, Hiroyuki Nishiyama

**Affiliations:** 1 Department of Urology, Institute of Medicine, University of Tsukuba, Tsukuba 305-8575, Japan; s2130369@s.tsukuba.ac.jp (K.S.); s2230394@u.tsukuba.ac.jp (S.S.); s2430392@u.tsukuba.ac.jp (R.T.); s2330386@u.tsukuba.ac.jp (B.I.); s2330437@u.tsukuba.ac.jp (R.Y.); hamadaskazuki@md.tsukuba.ac.jp (K.H.); tanuma.kozaburo.ge@u.tsukuba.ac.jp (K.T.); snitta@md.tsukuba.ac.jp (S.N.); kojou-tuk@md.tsukuba.ac.jp (K.K.); mshiga@md.tsukuba.ac.jp (M.S.); ynagumo@md.tsukuba.ac.jp (Y.N.); aikeda@md.tsukuba.ac.jp (A.I.); tkawahar@md.tsukuba.ac.jp (T.K.); a-hoshi@md.tsukuba.ac.jp (A.H.); nishiuro@md.tsukuba.ac.jp (H.N.); 2 International Medical Center, University of Tsukuba Affiliated Hospital, Tsukuba 305-8576, Japan; bmathis@md.tsukuba.ac.jp; 3 Graduate School of Nursing Science, St. Luke’s International University, Chuo-ku, Tokyo 104-0044, Japan; okuyama.ayako.r3@slcn.ac.jp; 4 Division of Health Services Research, National Cancer Center Institute for Cancer Control, Chuo-ku, Tokyo 104-0045, Japan

**Keywords:** penile cancer, squamous cell carcinoma, extramammary Paget disease

## Abstract

**Background/Objectives**: Penile malignant tumors are rare neoplasms, and both clinicopathologic features and prognoses remain unclear in Japanese patients. Here, we investigated the prognoses of penile malignant tumors in Japan using the nationwide hospital-based cancer registry (HBCR) database. **Methods**: The 2015 HBCR database cohort was queried to identify patients with penile malignant tumors. Moreover, we investigated age, pathology, tumor–node–metastases classification, first-course treatment, and overall survival in these patients. **Results**: A total of 269 patients were analyzed from the 2015 cohort (149 squamous cell carcinoma [SCC] patients and 56 extramammary Paget disease [EMPD] patients). The median ages at diagnosis in both SCC and EMPD patients were 76.0 and 76.5 years. The median observation period was 53 months in the present study. The 5-year overall survival (OS) rates of SCC and EMPD were 56.8% and 78.5%. The 5-year OS rates of clinical stages 0–I, II, III, and IV were (respectively) 68.0%, 70.1%, 38.5%, and 11.9% in SCC patients and (respectively) 83.7%, 83.3%, not evaluated, and 0% in patients with EMPD. **Conclusions**: We revealed the OS of Japanese patients with penile SCC and EMPD in a large population-based study for the first time. Patients with clinical stage IV penile SCC and EMPD had extremely poor prognoses, highlighting a need for improved disease management in these patients.

## Simple Summary

Penile malignant tumors are rare, and their clinical outcomes in Japanese patients remain poorly understood. Using the hospital-based cancer registry database in Japan, we analyzed 205 patients diagnosed in 2015, including 149 with squamous cell carcinoma (SCC) and 56 with extramammary Paget disease (EMPD). The median age at diagnosis was approximately 76 years for both groups. The 5-year overall survival rates were 56.8% for SCC and 78.5% for EMPD. Survival outcomes worsened markedly with advanced clinical stage. In SCC patients, the 5-year overall survival rates were 68.0% for stage 0–I, 70.1% for stage II, 38.5% for stage III, and 11.9% for stage IV disease. Patients with stage IV EMPD also had extremely poor outcomes. This study provides the first large-scale survival data for Japanese patients with penile SCC and EMPD and highlights the need for better treatment strategies for advanced disease.

## 1. Introduction

Penile cancer is a rare neoplasm, with a global estimated age-standardized incidence rate (ASIR) of 0.8 per 100,000 men in 2020 [1]. The incidence has been increasing in many areas in the past few decades. However, the trends in penile cancer incidence varied across countries [1]. There is a significantly increasing trend in the ASIR of penile cancer among most European countries, especially in the United Kingdom, Lithuania, Norway, Estonia, and Cyprus. Similarly, the ASIR has increased in China and Israel. Conversely, the ASIR has decreased in the Philippines, India, the USA, Canada, and Japan. Thus, one feature is the considerable geographic variation in disease incidence.

The majority of penile cancers are squamous cell carcinomas (SCCs), which are pathologically classified into two categories: HPV-associated SCCs (basaloid squamous cell carcinoma, warty carcinoma, clear cell carcinoma, lymphoepithelioma-like carcinoma, and mixed carcinoma) and HPV-independent SCCs (usual type, verrucous carcinoma, papillary squamous cell carcinoma, sarcomatoid carcinoma, and mixed carcinoma) [2]. The frequency of usual-type SCC is reported to be approximately 70–75% [3]. Moreover, the WHO 2022 classification listed adenocarcinoma, adenosquamous carcinoma, and extramammary Paget disease (EMPD) as other carcinomas of the penis [2].

Penile SCC is considered either as a malignant transformation of penile intraepithelial neoplasia or de novo, possibly in response to the presence of risk factors [3]. Several risk factors have been suggested, including poor genital hygiene, lack of circumcision, HPV infection, smoking, and other lifestyle factors. A meta-analysis revealed that the HPV pooled prevalence estimates for Eastern and South-Eastern Asia were half those of other regions in males [4]. As a result of its rarity, no studies have investigated the prognosis of penile SCC patients using large-scale data in Japan.

EMPD is also a rare intraepidermal adenocarcinoma arising either as a primary tumor or by secondary involvement from an underlying malignancy in the genitourinary or gastrointestinal tract [2]. Lindsey et al. reported that the age-adjusted incidence rate was 3.7 per million persons per year for Asian men, based on analysis of the Surveillance, Epidemiology, and End Results (SEER) database [5]. The incidence is fourfold higher in Asian compared to non-Asian men [5] and commonly occurs in the scrotum, penis, perineum, perianal area, and/or pubic area [6]. Penile EMPD accounts for only 6.8% of reported cases of male EMPD [7]; therefore, penile EMPD is extremely rare and, as such, lacks well-reported clinical information, including prognosis data.

The Japanese hospital-based cancer registry (HBCR) is a nationwide registry system for cancer in which designated cancer care hospitals (DCCHs) routinely collect clinical data for the HBCR database, which covers ~67% of newly diagnosed cancer patients in Japan [8]. DCCHs serve as regional centers for cancer care, providing highly specialized medical services to patients while also offering training opportunities for healthcare professionals. To maintain the quality and accuracy of the registry, participating hospitals are required to employ at least one tumor registrar who has successfully completed a basic training program provided by the National Cancer Center. The National Database of the HBCR provides an overall picture of cancer care in Japan.

In the present study, we investigated the clinical features of penile malignancies in the 2015 HBCR cohort. Moreover, we revealed the prognoses of penile SCC and EMPD in Japan.

## 2. Materials and Methods

### 2.1. Data Source

The HBCR includes both demographic and cancer characteristic data (including the topology and morphology codes of the International Classification of Diseases for Oncology, Third Edition [ICD-O-3]) [9], the TNM stages according to the 7th edition of the TNM Classification of the Union for International Cancer Control (UICC) [10], and initial treatments.

### 2.2. Selection of Data

We collected HBCR data for penile tumor patients diagnosed in 2015. The penile tumor cases were identified using topography codes C60.0 (Prepuce), C60.1 (Glans penis), C60.2 (Body of penis), C60.8 (Overlapping lesion of penis), and C60.9 (Penis, NOS), plus morphology codes drawn from the ICD-O-3 codes. In the present study, we selected only those patients who started first-course treatments at participating hospitals to avoid duplicate counts.

### 2.3. Pathology and Classification

The pathological diagnosis was classified based on the WHO 2022 classification of penile cancers [2]. Usual type (8070/2 to 8073/3), papillary squamous cell carcinoma (8050/3), warty carcinoma (8051/3), verrucous carcinoma (8051/3), sarcomatoid carcinoma (8074/3), lymphoepithelioma-like carcinoma (8082/3), basaloid squamous cell carcinoma (8083/3), and clear cell squamous cell carcinoma (8084/3) were classified as SCC based on ICD-O codes. EMPD was defined exclusively by the ICD-O-3 code 8542/3.

### 2.4. Data Analysis

We described the age distribution, anatomical site, pathology, TNM classification, and treatment, then examined the patterns of first-course treatment stratified by clinical stage. Due to HBCR regulations on avoiding identification, it was impossible to unveil the exact number of patients if there were fewer than 10 cases for any particular group. The overall survival (OS) rate was analyzed by the Kaplan–Meier method using R (4.4.2 for Windows^®^, R Foundation [11]). All survival analyses were performed using the “survival” (version 3.7-0) and “survminer” (version 0.5.1) packages in R.

## 3. Results

### 3.1. Patient Eligibility

**Figure 1:**
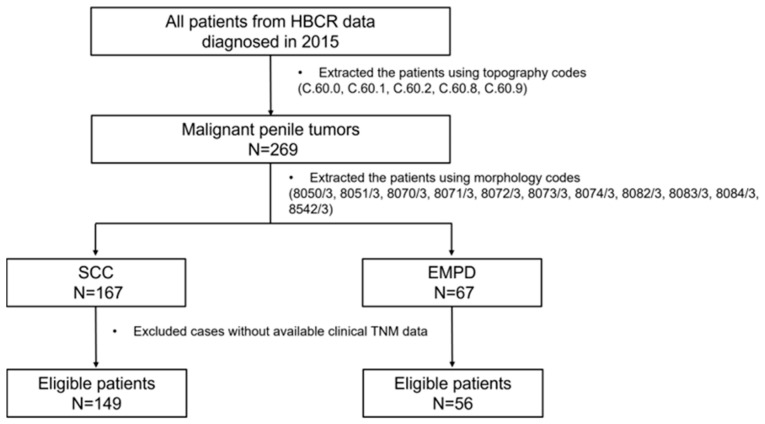
Eligibility of patients with penile malignancies in the 2015 cohort of the HBCR.

The eligibility flowchart of the present study is shown in Figure 1. Of 269 patients registered with malignant penile tumors from 164 hospitals in the 2015 cohort, 167 were histologically diagnosed with SCC, whereas 67 were diagnosed with EPMD. After exclusion of 29 patients due to missing values about clinical TNM data, we finally analyzed 149 SCC and 56 EMPD patients in the present study.

### 3.2. Patient Characteristics

SCC and EMPD patient characteristics are shown in Table 1. The median age at diagnosis was 76.0 years in patients with SCC and 76.5 years in patients with EMPD. In approximately half of the patients (54.4%), SCCs were located on the glans, followed by the shaft/cavernous body (13.4%) and foreskin (10.1%). On the other hand, lesions located on the glans or foreskin were not observed in patients with EMPD. However, the primary tumor location was unknown in 75% of the patients. EMPD patients had a higher incidence of localized disease (clinical stage 0, I, and II) compared to SCC patients (87.5% vs. 69.8%, respectively). Patients with metastatic disease were uncommon in both penile SCC and EMPD.

### 3.3. Overall Survival of Penile SCC and EMPD Patients

**Figure 2:**
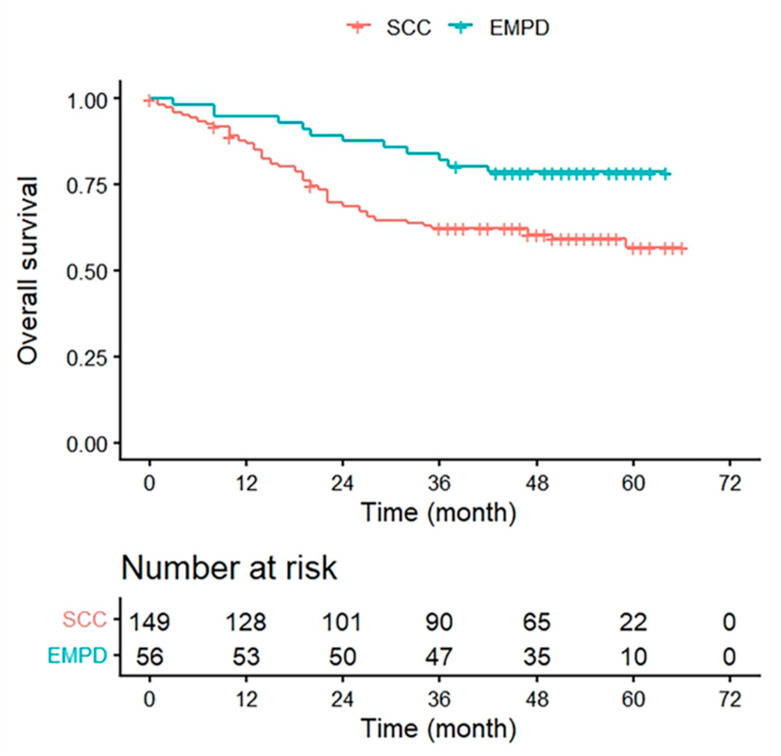
The overall survival in penile SCC and EMPD patients.

The median observation period was 53 months (SCC 52 months, EMPD 53 months) in the present study. The median OS was not reached in patients with SCC or EMPD (Figure 2). The 5-year OS rates of SCC and EMPD were 56.8% (95% confidence interval [CI]: 48.3–66.9) and 78.5% (95% CI: 68.5–90.1).

**Figure 3:**
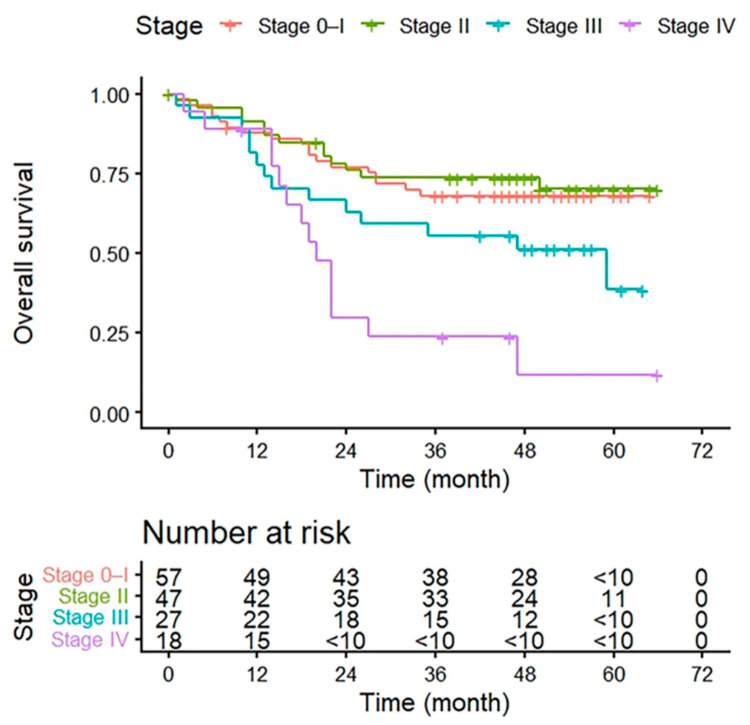
The overall survival in patients with penile SCC, according to clinical stage.

The 3-year OS rates for clinical stages 0–I, II, III, and IV were (respectively) 68.0% (95% CI: 56.8–81.4), 73.6% (95% CI: 61.9–87.6), 55.6% (95% CI: 39.6–77.8), and 23.7% (95% CI: 10.1–55.7) in patients with SCC (Figure 3). The 5-year OS rates for clinical stages 0–I, II, III, and IV were (respectively) 68.0% (95% CI: 56.8–81.4), 70.1% (95% CI: 57.5–85.5), 38.5% (95% CI: 19.5–75.7), and 11.9% (95% CI: 2.3–60.4) in patients with SCC (Figure 3). Importantly, the median OS was 20 months in SCC patients with clinical stage IV.

**Figure 4:**
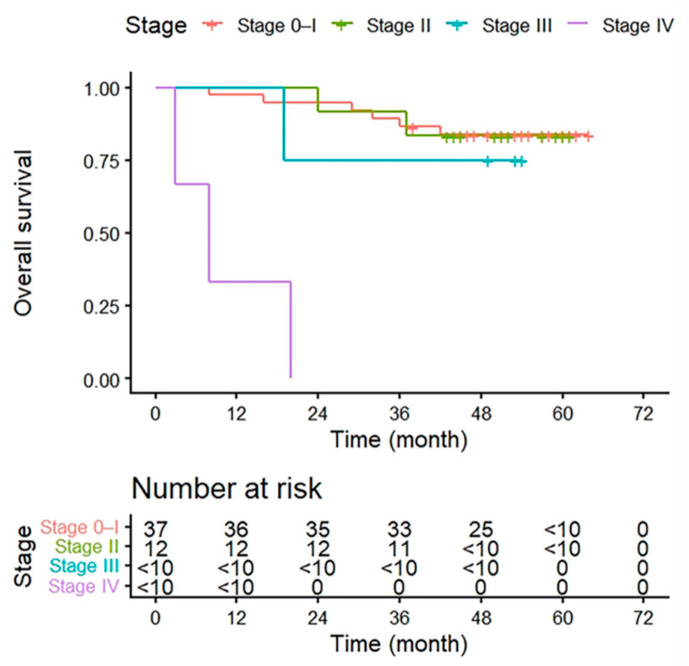
The overall survival in patients with penile EMPD, according to clinical stage.

On the other hand, the 3-year OS rates for clinical stages 0–I, II, III and IV were (respectively) 86.5% (95% CI: 76.1–98.2), 91.7% (95% CI: 77.3–100), 75.0% (95% CI: 42.6–100), and 0% (95% CI: NE-NE) in patients with EMPD (Figure 4). The 5-year OS rates for clinical stages 0–I, II, III, and IV were (respectively) 83.7% (95% CI: 72.6–96.5), 83.3% (95% CI: 64.7–100), not evaluated (NE), and 0% (95% CI: NE-NE) in patients with EMPD (Figure 4). Surprisingly, the median OS was 8 months in EMPD patients with clinical stage IV.

### 3.4. Patterns of First-Course Treatment for Penile SCC and EMPD Patients

The patterns of first-course treatments for SCC or EMPD according to clinical stage are shown in Table 2. Almost all SCC and EMPD patients without stage IV disease were treated with surgery, whereas radiotherapy was selected in only a few patients (fewer than 10 for each SCC stage and stage 0–I EMPD). In addition, only about half of stage IV SCC patients (55.6%) and fewer than 10 stage IV EMPD patients were initially treated with chemotherapy.

## 4. Discussion

Data on malignant penile tumor prognoses in Japan are limited. Yamada et al. reported the prognoses of 59 patients with penile cancer at Kobe University Hospital in 1998 [12], showing that the 5- and 10-year cause-specific survival (CSS) rates were 75.9% and 73.8%. Yamaguchi et al. also reported the prognoses of 196 patients with penile SCC in the Kyushu–Okinawa area of Japan who were treated from 2009 to 2020 [13]. The 5-year OS and CSS rates were, respectively, 74.3% and 79.8%, with a median follow-up of 26 months. However, these retrospective studies were conducted in university hospitals and their affiliated hospitals. To our knowledge, there is no large-scale, population-based report of survival outcomes for penile cancer in Japan. In the present study, we revealed the OS for Japanese patients with penile SCC and EMPD using data derived from the HBCR.

There are several extant, large population-based reports about penile SCC prognoses of patients in Western countries. Deng et al. reported that the 5-year relative survival rate of 6397 patients who were diagnosed with penile cancer between 2010 and 2014 was 65.7% in the United States, based on the SEER database [14]. Hanse et al. reported a 5-year relative survival rate of 61.6% among 1596 patients who were diagnosed with penile cancer from 2011 to 2015 using data from the Cancer Registry of Norway [15]. Moreover, Boehm et al. reported that the 5-year relative survival rate of 898 patients who were diagnosed with penile cancer from 2000 to 2018 was 74.7% according to a database in Saxony, Germany [16]. On the other hand, the 5-year OS rate of penile SCC patients was 56.8% in the present study. Although direct comparisons should be avoided, these differences might be due to heterogeneous backgrounds, such as age distribution, clinical or pathological stages, and comorbidities among each cohort, including the other Japanese cohort.

Contrary to expectations, the 5-year OS rates for patients with clinical stage 0–I penile SCC were slightly lower than those for patients with clinical stage II disease in the present study (68.0% vs. 70.1%). No significant difference in the rate of surgery was observed between the two groups. Because the cause of death was unavailable in this study, it is possible that, given the relatively small cohort size, the observed results were influenced by deaths from causes other than penile cancer.

The present study also revealed that penile SCC patients with advanced disease have poor prognoses, especially with regard to 5-year OS rates for clinical stage IV, as seen in the present study (11.9%). Graafland et al. also reported similar high-stage drops in relative OS rates from 1989 to 2006 by analyzing the national, population-based Netherlands Cancer Registry database [17]. In that study, the 10-year relative survival of patients with stage 0, I, and II tumors was 93%, 89%, and 81%, respectively; however, the 2-year relative OS was 21% in patients with stage IV. Moreover, Deng et al. revealed that the 5-year relative OS rates of advanced disease in the periods 2000–2004, 2005–2009, and 2010–2014 were (respectively) 17.29%, 14.75%, and 15.56% in the United States [14]. These results suggest that advanced penile SCC treatment management remains a global issue. Indeed, only 55.6% of patients were treated with chemotherapy in the present study, even in cases of stage IV disease.

We previously investigated patterns of first-course cancer treatment for 1012 penile SCC patients who were registered in the HBCR database between 2012 and 2015 [18]. Similar to the present study, only 44.1% of 93 patients with clinical stage IV underwent chemotherapy. Importantly, the proportion of patients who underwent chemotherapy was significantly lower in elderly patients (≥80 years) with clinical stage IV disease (53.2% vs. 14.3%). Although this is purely speculative due to a lack of clinical data, treatment tolerability and comorbidities may influence treatment selection in elderly patients with cancer. Therefore, there is a need for effective systemic therapies with fewer adverse events, because penile SCC patients are often elderly [19]. Interestingly, Zhu et al. evaluated the tumor microenvironment (TME) of penile SCC by performing single-cell transcriptomic profiling. This study showed that CD8^+^ T cells in HPV-positive penile SCC exhibited a less exhausted phenotype, whereas HPV-negative tumors were enriched for TIGIT and its ligands, suggesting a more immunosuppressive TME. Indeed, clinical trials have been conducted to evaluate immunotherapy, immunotherapy-based combination therapy, oncolytic vaccines, and antibody–drug conjugates [20,21,22,23,24,25]. Unfortunately, a triplet systemic chemotherapy regimen, including paclitaxel, ifosfamide, and cisplatin, is still recommended as the first-line systemic therapy in the National Comprehensive Cancer Network (NCCN) guideline [26]. It is hoped that innovative therapeutic strategies for metastatic penile SCC will be developed in the future.

In contrast to penile SCC, studies that focus on the prognosis of penile EMPD are scarcer. In fact, the most common site of EMPD was the vulva (57.7%), followed by truncal skin (19.7%), the scrotum (14.4%), the perianal area (3.2%), and the penis (2.7%) in the previous reports analyzing the SEER database [27]. Therefore, penile EMPD is extremely rare. Here, we revealed that the 5-year OS rate of Japanese patients with penile EMPD was 78.5% (median OS not reached). Although it is difficult to directly compare with our data, Weng et al. investigated the prognoses of 771 patients with EMPD using SEER data from 1973 to 2013 [27]. The disease-specific 5-year OS rate was 87% in all EMPD patients and, compared with perianal EMPD patients (median OS 113.5 months), patients with penile EMPD (HR 0.216, median OS 165.9 months) had better survival. These results suggest that not all cases of penile EMPD carry a poor prognosis.

Similar to penile SCC, penile EMPD patients with clinical stage IV disease also had poor prognoses in the present study. Weng et al. reported that the median OS of localized, regional, and distant EMPD was 346.0, 283.3, and 46.6 months, respectively [27]. Moreover, Ohara et al. revealed that patients with distant metastasis had a 5-year survival rate of only 7%, but this rose to 84% for those without metastasis in the Japanese EMPD cohort [28]. However, even with such a low survival rate, no consensus on the optimal systemic therapy for metastatic EMPD has yet been reached [6,29]. The evidence of systemic therapy for metastatic EMPD is extremely limited. Although there are small retrospective studies, the overall response rate (ORR) of heterogeneous chemotherapy regimens was 59.6% [29]. In two retrospective studies, patients who received systemic therapy showed a trend toward improved overall survival (OS) compared with those who received best supportive care; however, the difference did not reach statistical significance. Therefore, there is an urgent need for the development of effective therapeutic strategies that can improve the prognosis of patients with metastatic EMPD. Recently, human epidermal growth factor receptor 2 (HER2) has been focused on as a therapeutic target for metastatic EMPD, because HER2 gene amplification in the metastatic lesions was detected (37.1%) [30]. A phase II trial showed the ORR of a combination therapy with docetaxel plus trastuzumab for HER2-positive advanced EMPD was 76.9% [31]. The median OS was not reached at the median follow-up of 27.9 months. Further research is needed to establish the therapeutic strategy for HER2 in patients with metastatic EMPD.

The HBCR system is the first nationwide unified cancer registry system implemented in Japan for all cancer types. Because the number of DCCHshas increased and non-designated hospitals have been included in the national HBCR database since 2011, the number of hospitals participating in the HBCR has gradually risen. Consequently, the overall HBCR coverage reached 71.7% in 2017 [32]. Therefore, it is expected to further increase the number of registered cancer cases in Japan, leading to a greater accumulation of cases in all cancer types in the HBCR database. In the future, the HBCR system will be able to provide clinical characteristics and prognosis in several types of rare cancer patients in Japan, using a larger cohort with long follow-up data. Consequently, it would bring evidence for clinical practice for rare cancers.

There are several inherent limitations to the present study due to HBCR data availability. First, the population was relatively small because malignant penile tumors are rare, and available data are limited to patients diagnosed only in 2015. Second, no detailed information about the clinical condition (i.e., cause of death) was available from the HBCR, potentially affecting the analysis of survival outcomes in the present study. Third, we do not have detailed data on first-course treatments (i.e., surgical procedures chosen or chemotherapy regimens). Fourth, the present study primarily includes data from DCCHs, which serve as central providers of cancer care within their respective communities and regions. Fifth, we were unable to report the numbers of some subgroups due to HBCR regulations. This limitation may have constrained the interpretation of the subgroup analyses. Sixth, tumor stage was classified according to the UICC TNM Classification, 7th edition, which was the standard staging system during the study period. Because the 8th edition has been adopted since 2017, differences between the two staging systems may limit direct comparisons with more recent studies. Seventh, the survival estimates were not directly comparable because our cohort showed overall survival, whereas the cancer registry databases of other countries provided relative survival, which is adjusted for expected mortality in the general population. Finally, at the time of manuscript preparation, HER2-targeted therapies have not yet been approved for EMPD in Japan, precluding analysis of any effect on the study population.

Therefore, it is possible that patients with penile SCC or EMPD are treated differently in DCCHs compared to community hospitals. Despite these limitations, the present study reveals important information regarding rare penile SCC and EMPD in Japan.

## 5. Conclusions

We revealed the prognoses of Japanese patients with penile SCC and EMPD in a large, population-based study for the first time. The BHCR registry supports to reveal clinical characteristics and prognosis of rare cancers in Japan. In fact, prognoses for both penile SCC and EMPD patients with clinical stage IV were extremely poor, and improvement of disease management for such patients is expected in the future.

## Figures and Tables

**Table 1:** Patient characteristics between penile SCC and EMPD patients.

	SCC	EMPD
**Median age, years (range)**	76.0 (33–93)	76.5 (55–91)
**Anatomical site, *n* (%)**		
Foreskin	15 (10.1)	0 (0)
Glans	81 (54.4)	0 (0)
Shaft and cavernous body	20 (13.4)	14 (25.0)
Unknown	33 (22.1)	42 (75.0)
**Clinical T stage, *n* (%)**		
Ta/Tis	1–9	1–9
T1	65 (43.6)	35 (62.5)
T2	50 (33.6)	10 (17.9)
T3	28 (18.8)	1–9
T4	1–9	1–9
**Clinical N stage, *n* (%)**		
N0	104 (69.8)	50 (89.3)
N1	12 (8.0)	1–9
N2	18 (12.1)	1–9
N3	15 (10.1)	1–9
**Clinical M stage, *n* (%)**		
M0	140–148	47–55
M1	1–9	1–9
**Clinical stage, *n* (%)**		
Stage 0–I	57 (38.3)	37 (66.1)
Stage II	47 (31.5)	12 (21.4)
Stage III	27 (18.1)	1–9
Stage IV	18 (12.1)	1–9

**Table 2:** Patterns of first-course treatment in penile SCC and EMPD patients.

	Treatment	Stage 0–I	Stage II	Stage III	Stage IV
**SCC**	Surgery, *n* (%)	52 (91.2)	44 (93.6)	26 (96.3)	12 (66.7)
	Radiation, *n* (%)	1–9	1–9	1–9	1–9
	Chemotherapy, *n* (%)	1–9	1–9	1–9	10 (55.6)
**EMPD**	Surgery, *n* (%)	37 (100)	11 (91.7)	1–9	1–9
	Radiation, *n* (%)	1–9	0	0	0
	Chemotherapy, *n* (%)	0	0	0	1–9

## Data Availability

Due to data use agreements, the authors are not permitted to share the dataset.

## Abbreviations

The following abbreviations are used in this manuscript:

ASIR	Age-standardized incidence rate
SCC	Squamous cell carcinoma
EMPD	Extramammary Paget disease
HBCR	Hospital-based cancer registry
DCCH	Designated cancer care hospital
UICC	Union for International Cancer Control
ICD-O-3	International Classification of Diseases for Oncology, Third Edition
OS	Overall survival
CSS	Cause-specific survival
CI	Confidence interval
NE	Not evaluated
TME	Tumor microenvironment
NCCN	National Comprehensive Cancer Network
HER2	Human epidermal growth factor receptor 2
ORR	Overall response rate
